# Preoperative scoring system for the prediction of risk of lymph node metastasis in cervical cancer

**DOI:** 10.1038/s41598-024-74871-x

**Published:** 2024-10-11

**Authors:** Mu Xu, Xiaoyan Xie, Liangzhi Cai, DaBin Liu, Pengming Sun

**Affiliations:** 1 College of Clinical Medicine for Obstetrics & Gynecology and Pediatrics, Fujian Medical University, Fujian, China; 2 Department of Gynecology, Fujian Maternity and Child Health Hospital, Affiliated Hospital of Fujian Medical University, No. 18 Daoshan Road, Fuzhou, 350001 Fujian China; 3 Laboratory of Gynecologic Oncology, Fujian Maternal and Child Health Hospital, Affiliated Hospital of Fujian Medical University, No. 18 Daoshan Road, Fuzhou, 350001 Fujian China

**Keywords:** Cervical cancer, Lymph node metastasis, Risk factor, Scoring system, Prognosis, Cancer, Oncology, Risk factors

## Abstract

The study aimed to develop and validate a preoperative scoring system to predict the risk of lymph node metastasis (LNM) in cervical cancer (CC). A total of 426 stage IB1–IIA1 CC patients were randomly divided into two sets. A logistic regression model was used to determine independent factors that contribute to LNM. A preoperative scoring system was developed based on beta (β) coefficients. An area under the receiver operating curve (AUC) was used to test for model discrimination. Five-year overall survival (OS) rate was 91.7%. Multivariable logistic regression analysis showed that FIGO stage, tumor size, depth of invasion on MRI, and squamous cell carcinoma antigen levels were independent risk factors in the development set (all P < 0.05). The AUCs of the scoring system for the development and validation sets were 0.833 (95% CI = 0.757–0.909) and 0.767 (95% CI = 0.634–0.891), respectively. Patients who scored 0–2, 3–5, and 6–8 were classified into low-risk, medium-risk, and high-risk groups. Predicted rates were in accord with observed rates in both sets. The 5-year OS rates of the new groups were also significantly different for the entire group, development set, and validation set (all P < 0.05). LNM affects the prognosis of CC patients. The scoring system can be used to assist in evaluating the risk of LNM in CC patients preoperatively. It is easy to obtain and can provide reference for clinical treatment decision-making.

## Background

Cervical cancer (CC) is a common malignant tumor of the female reproductive system and the fourth most diagnosed cancer among women worldwide^1,2^. Lymph nodes (LN) status is a critical predictor of survival that has been applied to guide clinical treatment in CC patients^3,4^. The median 5-year overall survival (OS) rate of cervical cancer patients with and without lymph node metastasis (LNM) was < 80% and > 80%, respectively^5^. According to FIGO staging (2018)^6,7^, LNM is classified as stage IIIC, regardless of size or parametrial invasion, and requires concurrent chemoradiotherapy, which fully explains the importance of LNM. Therefore, accurate evaluation of preoperative LNM is crucial for developing individualized treatment, improving prognosis, and reducing mortality^8^. The LN status is mainly assessed based on preoperative pelvic magnetic resonance imaging (MR) or computed tomography (CT) examination^9–11^. However, the sensitivity of these tests is only 54–58%, and it is especially difficult to identify LNs without enlargement. Compared with MRI and CT, positron emission tomography- computed tomography (PET-CT) is relatively more accurate, with sensitivity up to 76–86%^12,13^. Owing to limited spatial resolution, it is difficult to distinguish them from inflammatory LNs. Moreover, the high cost and radiation exposure limit PET-CT’s wide clinical application.

Therefore, the purpose of this study was to develop a scoring system to predict the risk of LNM in patients with CC, based on clinical indicators that are simple and easy to obtain.

## Materials and methods

### Study population

**Fig. 1:**
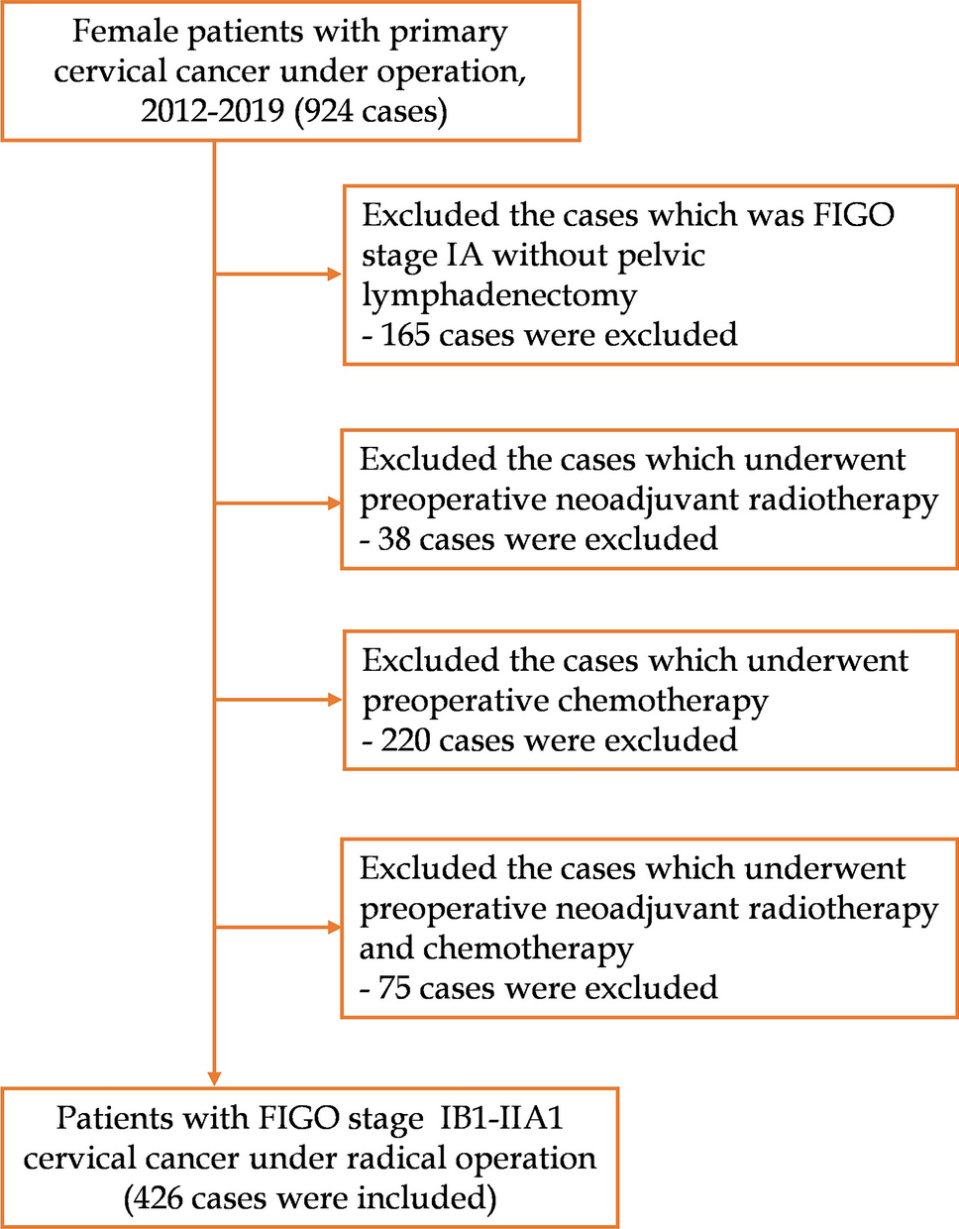
Inclusion and exclusion criteria for patients included in this study.

CC patients from the Fujian Maternity and Child Health Hospital between January 2012 and December 2019 were enrolled in the retrospective study. Since MRI is superior to CT in soft tissue resolution and can more objectively reflect tumor size and function, NCCN guidelines suggest that MRI examination is the first choice for cervical cancer^14^. Therefore, the enrolled patients were those who underwent MRI examination before surgery. Inclusion criteria included the following: (1) histologically confirmed CC without a fertility requirement; (2) patients diagnosed as FIGO stage IB1-IIA1; (3) radical hysterectomy with pelvic lymph node dissection was the first course of treatment; (4) CC was the only malignancy; and (5) preoperative pelvic MRI examination was performed routinely. Exclusion criteria included the following: (1) patients underwent neoadjuvant therapy; (2) patients with incomplete/inaccurate medical records (Fig. 1). FIGO staging criteria (2018) were used for tumor staging (Patients before 2018 were re-staged according to the gynecological description). Supplementary therapy was performed in patients with high-risk factors according to the postoperative pathological examination results. The study was approved by the Ethics Committee of the Fujian Provincial Maternity and Children’s Hospital (2023KY142). All methods were performed in accordance with the relevant guidelines and regulations as well as in compliance with the requirements of the Declaration of Helsinki.

### Follow-up

Patients with CC were followed up regularly after a radical surgery. The last follow-up date was March 31, 2023. The median follow-up period was 65.4 (range, 3.3–98.0) months. The patients were followed up every 3 months for the first 2 years after surgery, every 6 months during years 3–5 and then once a year until death or loss to follow-up. The follow-up information was obtained from hospital information systems and the patients or their relatives. OS was calculated from surgery to death or the last date of follow-up.

### Statistical analysis

All patients were divided into a development set and a validation set with a 2:1 ratio randomly. The data were statistically processed using SPSS for Windows version 26.0 (SPSS Inc., Chicago, IL, USA) and R × 64 ver. 4.2.2 (www.r-project.org). The patient characteristics were compared between groups using the chi-square test or Fisher’s exact probability method for categorical variables and a *t*-test or the Mann–Whitney U test for continuous variables. Overall survival outcome was analyzed with the Kaplan–Meier method and log-rank test. The logistic regression model was used to determine independent factors contributing to LNM. Potential indicators with P < 0.05 in the univariate analysis were introduced into the multivariate analysis. The scoring system was constructed by using risk predictors with P < 0.05. Each predictor in the multivariate model was associated with a β coefficient. The score was then achieved by dividing the β coefficient through the lowest β coefficient and rounding to the nearest integer^15^. The scores for each item were added together to calculate the total score for each patient. The area under the receiver operating curve (AUC) was presented as a statistical indicator for quantifying the discriminant ability of the system. The calibration was tested through the comparison between predicted (mean ± SD) and observed rates of LNM. The Hosmer–Lemeshow (H–L) test was used to evaluate the model fit. The trend of LNM among different groups was compared according to Sankey diagram, which performed using “ggalluvial” and “ggplot2” packages. P value < 0.05 was considered as statistically significant.

## Results

### Clinicopathological characteristics

In the study, a total of 426 CC patients were included, of whom 46 (10.8%) patients had LNM. There were 270 (63.4%) and 156 (36.6%) patients with a tumor size (TS) < 3 cm and ≥ 3 cm, respectively. The FIGO stages IB1, IB2, IB3, and IIA1 were observed in 189 (44.4%), 157 (36.8%), 25 (5.9%), and 55 (12.9%) patients, respectively. Invasion depths of < 1/2 and ≥ 1/2 of the stroma, as assessed by MRI (DI), were observed in 238 (55.9%) and 188 (44.1%) patients, respectively. There were 395 (92.7%) and 31 (7.3%) patients with normal or high Ca125 level, respectively. A total of 263 (61.7%) and 163 (38.3%) patients had normal and high SCC antigen levels, respectively. After random division, 284 and 142 patients were included in the development and validation sets, respectively. There was no statistically significant difference in the baseline data between the two groups (all P > 0.05) (Table 1). Otherwise, there were 17 patients were assessed LNM by MR before surgery, but only 9 of them were confirmed in pathology.

**Table 1:** Characteristics of patients in the development and validation sets.

Characteristics	Development set (N = 284)	Validation set (N = 142)	P value
Age(y)			0.364
< 45	111	62	
≥ 45	173	80	
BMI (kg/m^2^)			0.266
< 24	201	93	
≥ 24	83	49	
Histological type			0.154
Squamous	224	102	
Adenocarcinoma	46	34	
Adenosquamous	14	6	
Tumor size(cm)			0.670
< 3	178	92	
≥ 3	106	50	
FIGO Stage			0.421
Ib1	125	64	
Ib2	101	56	
Ib3	16	9	
IIa1	42	13	
Invasion by MRI			0.783
< 1/2	160	78	
≥ 1/2	124	64	
Ca125(U/ml)			0.147
< 35	267	128	
≥ 35	17	14	
SCC (ng/ml)			0.622
< 1.5	173	90	
≥ 1.5	111	52	
LNM			0.825
Negative	254	126	
Positive	30	16	

*BMI* body mass index, *FIGO* International Federation of Gynecology and Obstetrics, *MRI* magnetic resonance imaging, *Ca125* cancer antigen 125, *SCC* squamous cell carcinoma antigen, *LNM* Lymph node metastasis.

### Impact of LNM on survival outcomes

**Fig. 2:**
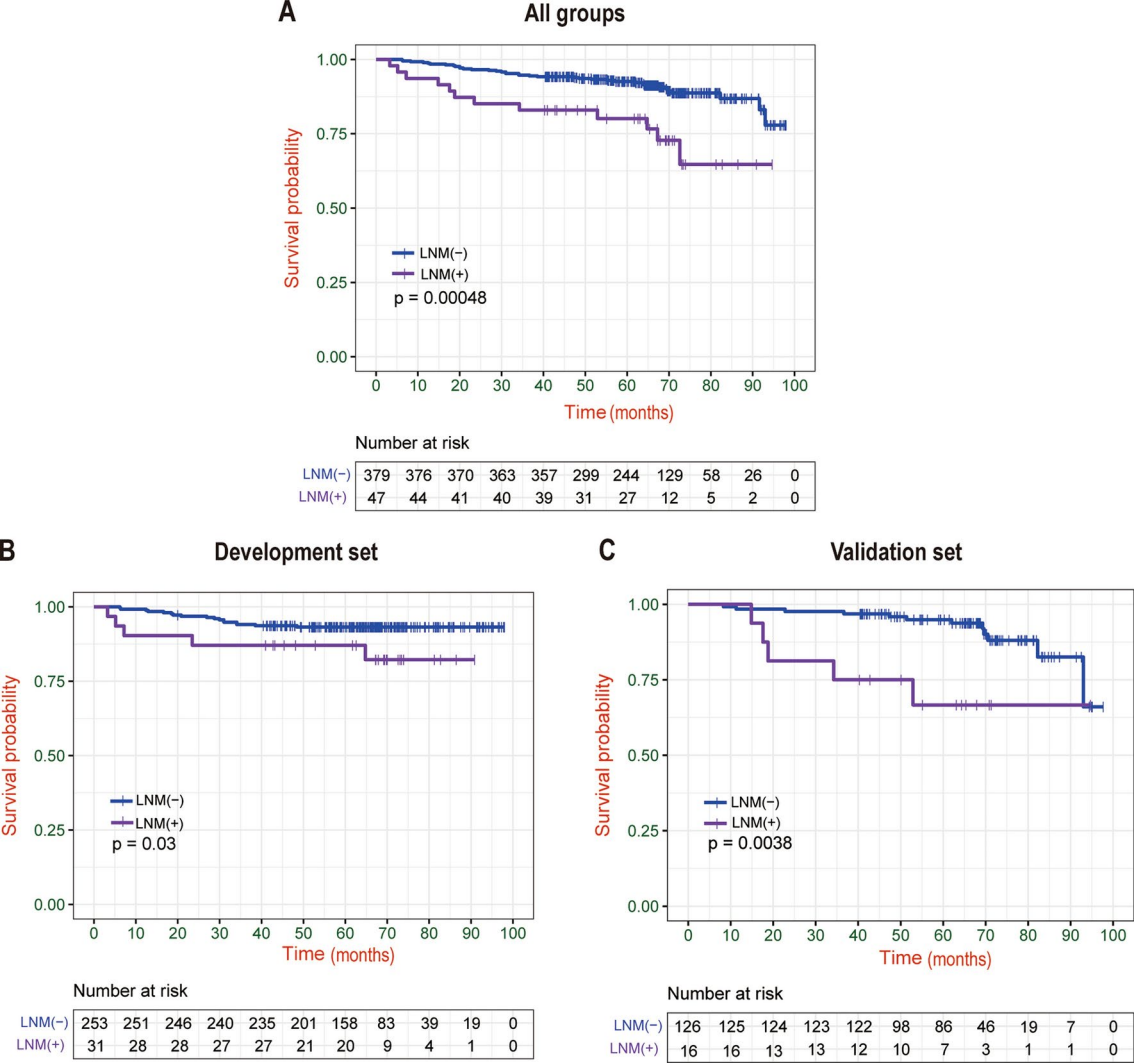
Kaplan–Meier survival curves stratified by LNM. In the (**A**) Entire group, (**B**) Development set, (**C**) Validation set.

The 5-year OS rate of all the patients and those with and without LNM were 91.7%, 79.1%, and 93.3%, respectively (P < 0.001). The difference in survival rate between the development and validation sets was also statistically significant (development set LNM negative vs. LNM positive 93.4% vs. 86.2%, P = 0.030; validation set LNM negative vs. LNM positive 95.5% vs. 66.7%, P = 0.003). (Fig. 2).

### Risk factor analysis of LNM in the development set

**Fig. 3:**
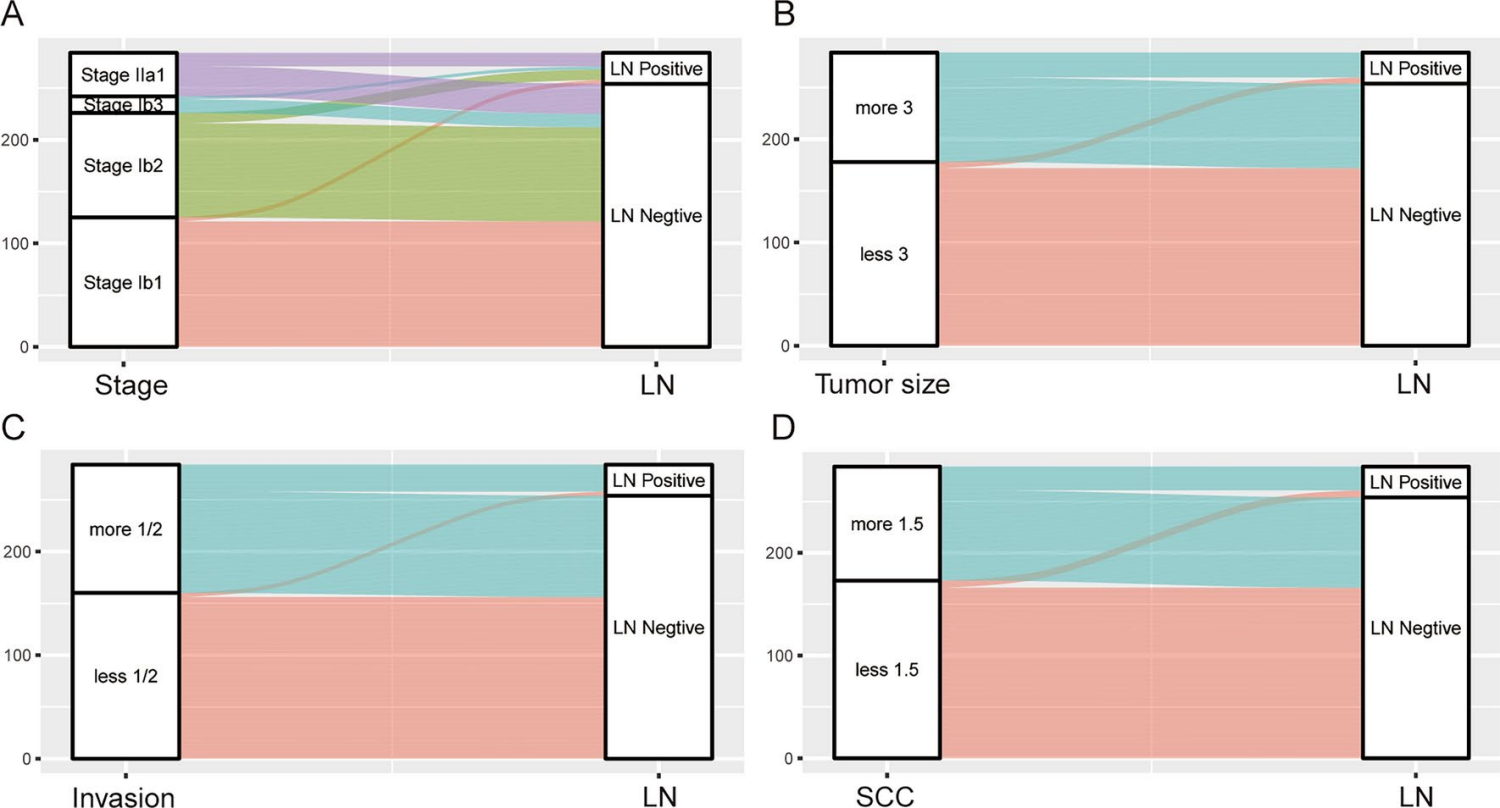
The Sankey diagram for the proportion of LNM in patients with different subsets. Stratified by (**A**) Stage. (**B**) Tumor size. (**C**) Tumor Invasion. (**D**) SCC antigen level.

Univariate analysis revealed that BMI, FIGO stage, TS, DI, and SCC antigen levels were associated with LNM (all P < 0.05). In a multivariate logistic regression analysis, FIGO stage, TS, DI, and SCC antigen levels were independent factors for LNM (Table 2). Additionally, the Sankey diagram vividly clearly shows the different proportions of LNM in patients stratified by FIGO stage, TS, DI, and SCC antigen levels (Fig. 3).

**Table 2:** Univariate and multivariate logistic regression analysis in the development set.

Characteristics	Univariate			Multivariate	
	LNM ( +)	LNM (-)	P value	OR (95% CI)	P value
Age(y)			0.195		
< 45	15	96		–	–
≥ 45	15	158			
BMI (kg/m^2^)			0.043		0.150
< 24	26	175		1	
≥ 24	4	79		0.408(0.120–1.383)	
Histological type			0.541		
Squamous	26	198		–	–
Adenocarcinoma	3	43			
Adenosquamous	1	13			
Tumor size(cm)			0.000		0.029
< 3	6	172		1	
≥ 3	24	82		6.319(1.213–32.923)	
FIGO Stage			0.000		0.020
Ib1	4	121		1	
Ib2	10	91		1.728(1.090–5.914)	
Ib3	3	13		4.492(1.464–13.77)	
IIa1	13	29		5.767(1.062–31.304)	
Invasion by MRI			0.000		0.020
< 1/2	4	156		1	
≥ 1/2	26	98		5.448(1.305–22.736)	
Ca125(U/ml)			0.868		
< 35	28	239		-	-
≥ 35	2	15			
SCC (ng/ml)			0.000		0.002
< 1.5	7	166		1	
≥ 1.5	23	88		5.782(1.91417.62)	

*BMI* body mass index, *FIGO* International Federation of Gynecology and Obstetrics, *MRI* magnetic resonance imaging, *Ca125* cancer antigen 125, *SCC* squamous cell carcinoma antigen.

### Risk scores

According to the β coefficient in the logistic regression model, each predictor was linked to a score (The baseline is 0, < 1 is 1, > 1 is 2). The detailed scores for each predictor are shown in Table 3. Then, the scores of each item were added up to calculate the total score. In this study, the lowest score was 0, and the highest score was 8.

**Table 3:** Scoring system.

Characteristics	β coefficient	Score
Tumor size(cm)		
< 3	–	0
≥ 3	1.843	2
FIGO Stage		
Ib1	–	0
Ib2	0.317	1
Ib3	1.502	2
IIa1	1.752	2
Invasion by MRI		
< 1/2	–	0
≥ 1/2	1.695	2
SCC (ng/ml)		
< 1.5	–	0
≥ 1.5	1.755	2

*FIGO* International Federation of Gynecology and Obstetrics, *MRI* magnetic resonance imaging, *SCC* squamous cell carcinoma antigen.

### Model accuracy and validity

**Fig. 4:**
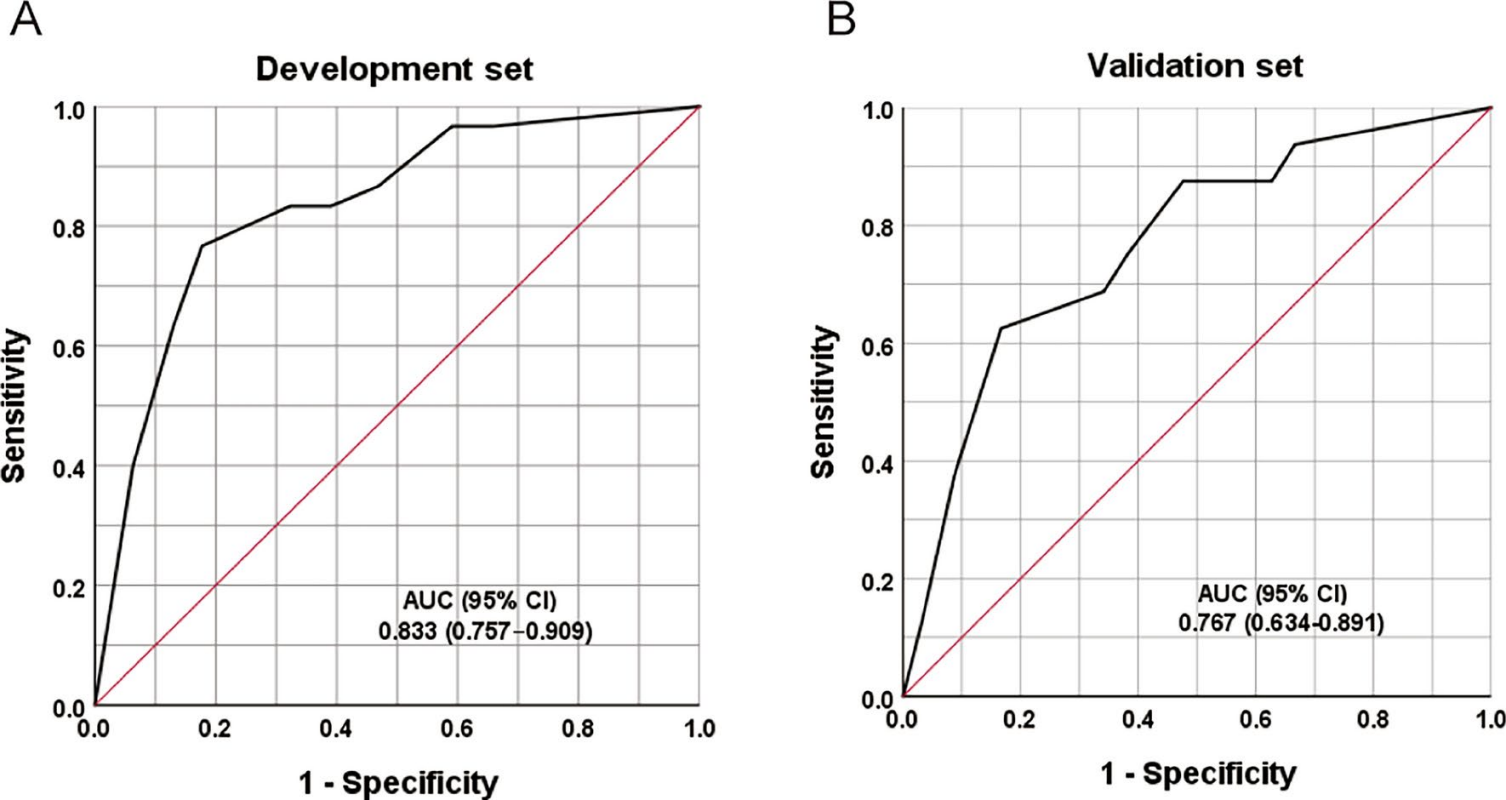
ROC curves in the (**A**) Development set and (**B**) Validation set.

**Fig. 5:**
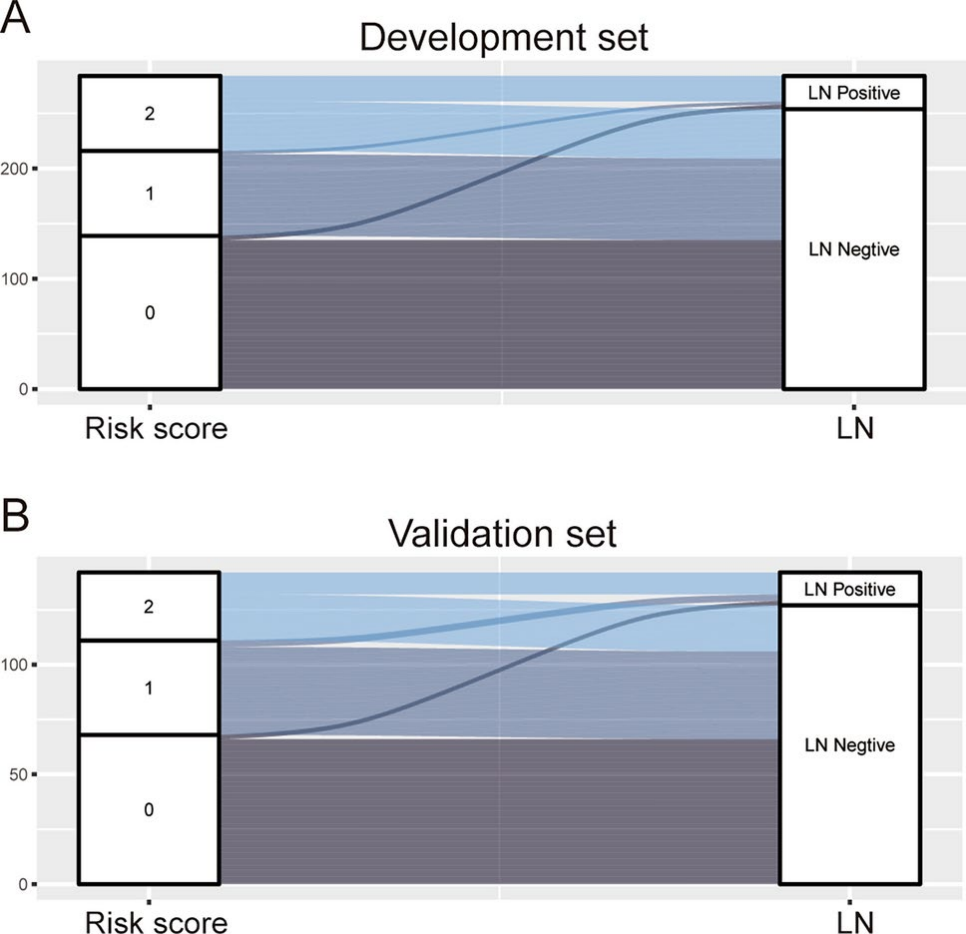
The Sankey diagram for the proportion of LNM in patients with different risk groups. (**A**) Development set and (**B**) Validation set.

**Fig. 6:**
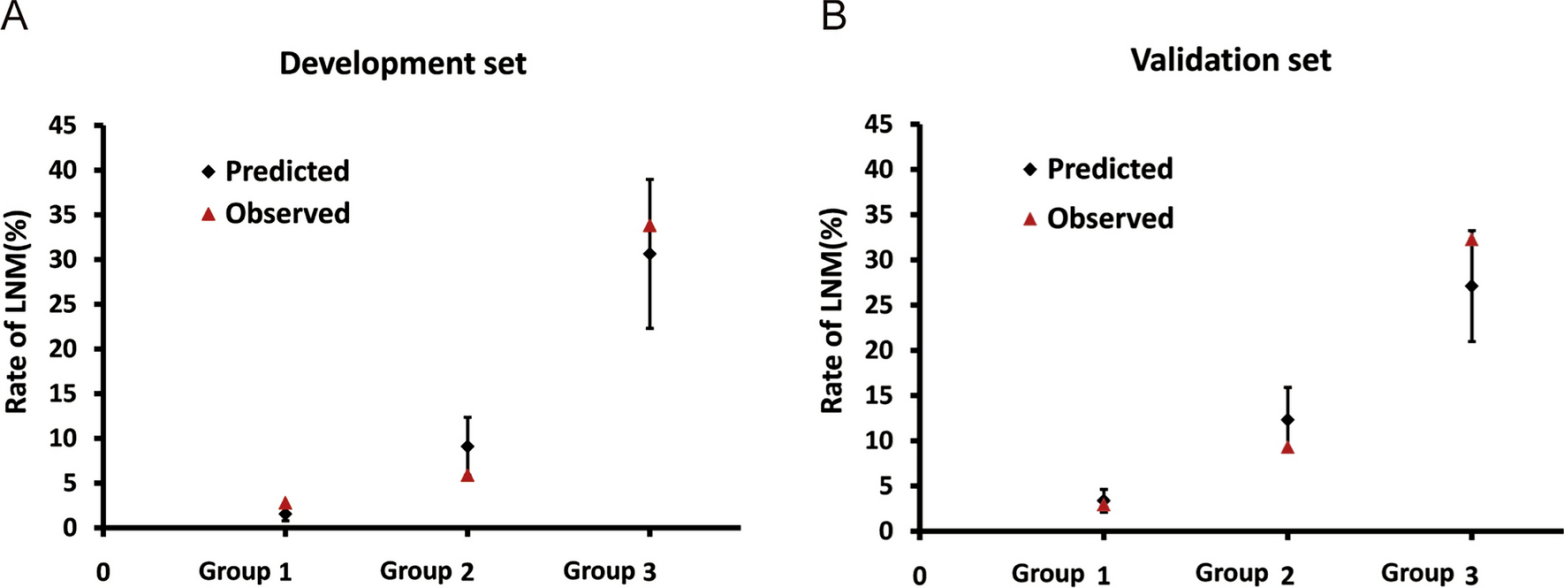
The predicted and observed rates of LNM (**A**) Development set and (**B**) Validation set.

The regression analysis indicated that an increase in score of 1 was associated with an increased risk of LNM by 1.681 times (95% CI = 1.386–2.039) for the development set and 1.447 times (95% CI = 1.138–1.841) for the validation set. The AUC for development and validation sets were 0.833 (95% CI = 0.757–0.909, P < 0.05) and 0.767 (95% CI = 0.634–0.891, P < 0.05) (Fig. 4), respectively. The H–L test for development (P = 0.218, x^2^ = 8.285) and validation sets (P = 0.259, x^2^ = 7.731) indicated that the model fit well. The risk classification was constructed based on the total scores. Patients scored 0–2 (Group 1), 3–5 (Group 2), and 6–8 (Group 3) were stratified into the low-, medium-, and high-risk groups, respectively. As shown in Fig. 5, the proportion of patients showed an increasing trend with the risk score increase in both development and validation sets. The predicted rates (mean ± SD) and observed rates were compared in the three risk groups. As was shown in Fig. 6, the predicted rates were in accord with the observed rates in both the development (Group 1: 1.54 ± 0.76 vs. 2.80; Group 2: 9.10 ± 3.27 vs. 5.89; Group 3: 30.65 ± 8.32 vs. 33.82) and validation (Group 1: 3.38 ± 1.26 vs. 2.94; Group 2: 12.32 ± 3.59 vs. 9.30; Group 3: 27.11 ± 6.14 vs. 32.26) sets.

### Difference in prognosis by new score group

**Fig. 7:**
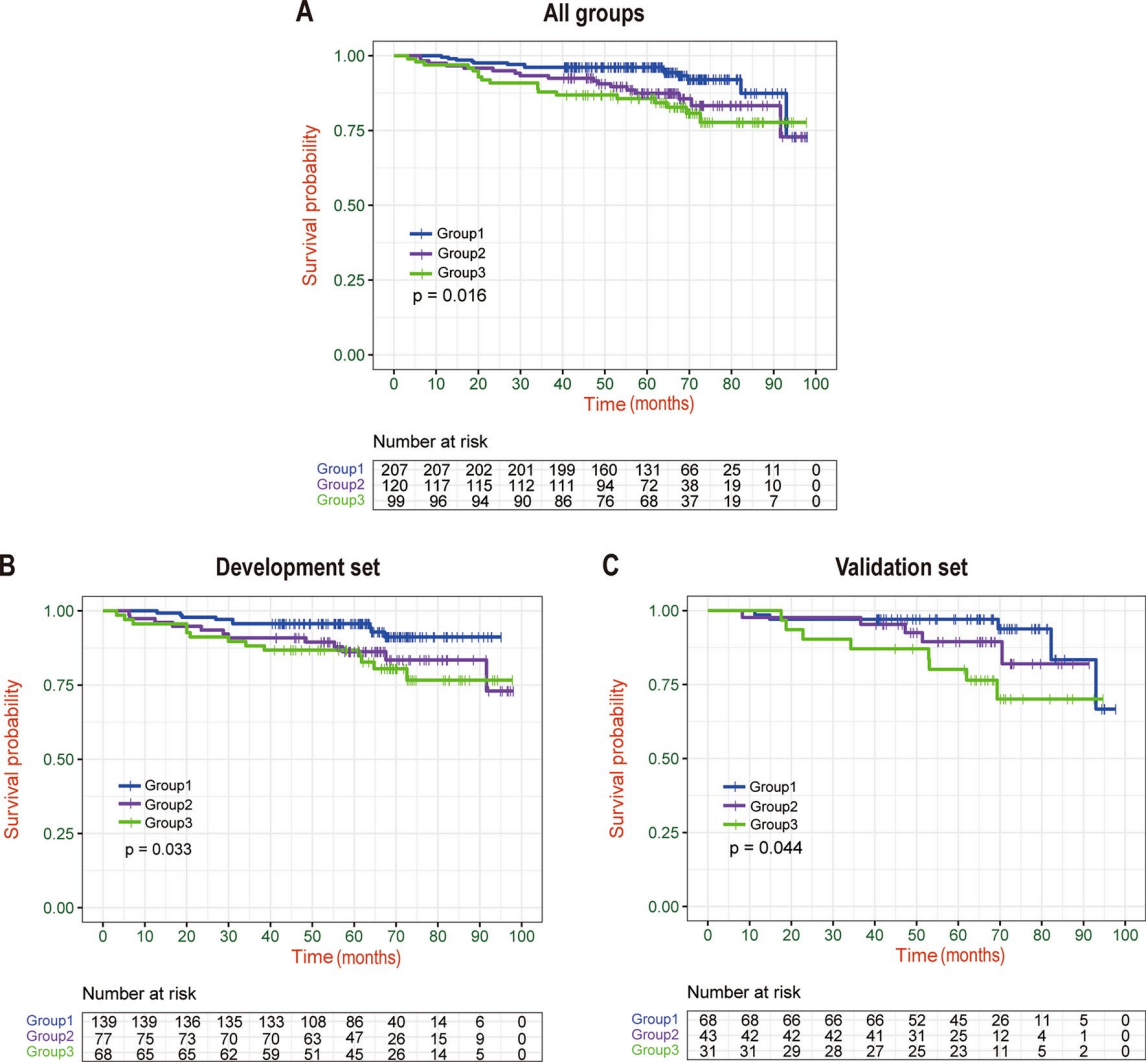
Kaplan–Meier survival curves stratified by new score group. In the (**A**) Entire group, (**B**) Development set, (**C**) Validation set.

Additionally, we compared the survival rates of the three groups using the new scoring system. The survival rates were statistically significant in the entire group (Group 1 vs. Group 2 vs. Group3: 96.02% vs. 89.95% vs. 85.37%, P = 0.016), development set (Group 1 vs. Group 2 vs. Group3: 95.58% vs. 88.90% vs. 86.76%, P = 0.033) and validation set (Group 1 vs. Group 2 vs. Group3: 96.92% vs. 91.34% vs. 83.05%, P = 0.044), respectively. (Fig. 7).

## Discussion

In our study, we had observed a statistically significant correlation between clinical indicators and LNM preoperatively. The correlation remained significant after multivariate analysis. Furthermore, we established a scoring system using the significant risk factors to predict the risk of LNM. This study reinforced the potential benefit of using preoperative clinical indicators to assess the risk of LNM in patients with CC.

Cervical cancer is a common gynecologic malignant tumor, and postoperative recurrence and metastasis are the main causes of death^16,17^. Lymph node metastasis, the main mode of metastasis in patients with CC, is a high-risk factor for recurrence and greatly impacts treatment and prognosis^3,4^. According to FIGO staging (2018)^6,7^, metastasis to LNs is classified as stage IIIC, regardless of size or parametrial invasion, and requires concurrent chemoradiotherapy. The study^18^ showed that in patients with LNM, the efficacy of chemoradiotherapy alone and surgery combined with chemoradiotherapy was equivalent (P = 0.765), whereas the efficacy of surgery alone was poor (P = 0.006). Therefore, accurate evaluation of preoperative LNM is crucial for developing individualized treatment, improving prognosis and reducing mortality^8^.

Currently, the LNM station in CC is mainly based on preoperative pelvic MRI or CT examination^9–11^, which is widely used in clinical practice. They are mostly assessed by measuring the size of LNs, resulting in a sensitivity of only 54–58%. Benedetti et al.^19^ found that malignancy was also possible when the diameter of the LN was < 1cm. Williams et al.^20^ also found that 54.4% of the metastatic LNs were < 1 cm in diameter. Moreover, the enlarged LNs may also be reactive hyperplasia but not metastases. Although size is an important indicator for evaluating the status of LN, it has limited value because of the overlapping sizes of benign and malignant LNs^21^. Therefore, routine preoperative pelvic MRI or CT examination makes it difficult to determine the status of metastasis based on LN size. Compared with MRI and CT, PET-CT is relatively more accurate, with a sensitivity of up to 76–86%^12,13^. Owing to PET-CT’s limited spatial resolution, high cost and high radiation dose, it is difficult to distinguish it from inflammatory LNs, which limits its clinical application^22,23^. In the study, most of the patients were assessed non-LNM by MRI pre-operation because of small diameter, resulting in a false negative. Therefore, we aimed to evaluate the risk of LNM by analyzing clinical indicators and constructing a scoring system.

This study showed a 10.8% rate of LNM in patients with FIGO stage IB1-IIA1 CC, in accordance with other studies^24,25^. Multivariable logistic regression analysis showed that FIGO stage, TS, DI, and SCC antigen levels were independent factors for LNM, which is also consistent with previous reports^26–28^. The new FIGO staging includes LN status, thereby indicating that LN status is of great significance for the treatment of patients, which affected the prognosis of CC^6^. Studies have shown that CC metastasis is correlated with staging. The higher the stage, the more likely it is that LNM will occur^29^. Invasive growth and metastasis are the main characteristics of malignant tumors. The larger the tumor diameter and the deeper the musculature invasion, the more likely the tumor cells will invade the intravascular system; thus, the more likely the development of LNM^23,25^. The SCC antigen is a specific serum tumor marker for CC that was first discovered by Kato in 1977^30^. The higher the SCC antigen level, the more aggressive the tumor and the higher the probability of LNM^17,31^. Studies^32,33^ have indicated that preoperative SCC value of patients is correlated with lymph vascular invasion, LNM, FIGO stage, and degree of tissue differentiation (P < 0.05). In this study, for patients with a higher FIGO stage, TS ≥ 3cm, invasion depth ≥ 1/2, and increased SCC level, the probability of LNM significantly increased, which was consistent with previous studies.

Subsequently, we assessed the weight of each indicator and constructed a scoring system (range, 0–8 points) to stratify CC patients with a different risk level of LNM. The AUCs of the proposed scoring system for the development and validation sets were 0.833 (95% CI = 0.757–0.909) and 0.767 (95% CI = 0.634–0.891), respectively, indicating the favorable discrimination ability of the model to accurately predict the risk of LNM for patients with CC. The H–L test for both sets also demonstrated that the model had no significant lack of fit. Moreover, patients were stratified into low-, medium-, and high-risk groups based on their scores. Calibration showed satisfactory consistency between the observed and predicted rates in development and validation sets. According to Sankey diagram, LNM trends were similar among the new groups. Additionally, we verified the 5-year survival rate of the new group, which was statistically significant in the entire group, development set, and validation set. The findings of this study have significant implications for clinical practice. Currently, studies on LNM based on imaging omics have received extensive attention^34–36^. However, deficiencies exist, such as insufficient standardization of medical image data and insufficient model generalization ability, which result in systematic deviations, poor biological interpretability, and poor clinical accessibility. In this study, we developed a scoring system based on relevant preoperative clinical indicators to preliminarily assess the LNM risk. These indicators avoid the interference caused by normal-sized or inflammatory swollen LNs, and are easy to obtain, making it easier for clinicians to make decisions. For high-risk patients, further examination should be performed before surgery, if necessary.

This study has some limitations. First, it was a retrospective design, which inevitably leads to observer bias and confusion. Second, the indicators used in the study were limited, and adding new factors may have increased the accuracy of the results. Third, the research was conducted at a single center with small number of cases, and multicenter data are needed to improve persuasion.

## Conclusion

In conclusion, we developed and validated a scoring system to stratify patients with CC at different risks for LNM before surgery. This model is beneficial for surgeons to make better clinical decisions and provides a reference for them, which has important clinical guiding significance.

## Data Availability

The datasets generated and/or analyzed during the current study are not publicly available due to personal information protection, patient privacy regulation, and medical institutional data regulatory policies, etc., but are available from the corresponding author on reasonable request.

## Abbreviations

BMI: Body mass index
FIGO: International Federation of Gynecology and Obstetrics
MRI: Magnetic resonance imaging
Ca125: Cancer antigen 125
SCC: Squamous cell carcinoma antigen
LNM: Lymph node metastasis
